# Integrity matters in oncology: AORTIC takes a stand against research misconduct

**DOI:** 10.1038/s44276-023-00032-8

**Published:** 2024-02-12

**Authors:** Khalid El Bairi, Dario Trapani, Laure-Anne Teuwen, Belmira Rodrigues, Miriam Mutebi

**Affiliations:** 1 Faculty of Medical Sciences, Mohammed VI Polytechnic University, Ben Guerir, Morocco; 2 European Institute of Oncology, IRCCS, 20141 Milan, Italy; 3 Department of Oncology and Hemato-oncology (DIPO), University of Milan, Milan, Italy; 4 Department of Oncology, Antwerp University Hospital, Edegem, Belgium; 5 African Organization for Research and Training in Cancer, Cape Town, South Africa; 6 Department of Surgery, Aga Khan University, Nairobi, Kenya

## Abstract

Oncology is a rapidly evolving field globally, with a growing need to maintain research integrity. The African Organization for Research and Training in Cancer (AORTIC) has initiated a comprehensive training program to enhance research quality and integrity in African oncology. This program covers topics such as predatory publishing, data manipulation, plagiarism, “paper mills,” gender equity, and the critical appraisal of clinical trials and meta-analyses beyond statistical significance. This emphasizes the importance of ethical conduct and responsible research in enhancing cancer care through research. The commitment of the AORTIC serves as a model for other oncology societies in low- and middle-income countries, highlighting the importance of education and training to reduce disparities in cancer research and empower African researchers.

## To the Editor:

Cancer research is constantly evolving, with the ultimate aim of enhancing care of patients with cancer and their outcomes globally. Assuring research integrity is essential in such a rapidly growing landscape, accompanying the major milestones of modern oncology. Cancer research has traditionally lagged behind biomedical research [1] but there is a critical need to increase not only the quantity but also the quality of outputs on the African continent. Amidst perverted logic behind some research conduct, focused primarily on careerism and profits, enhancing research integrity can mitigate the adverse effects of the “publish or perish” phenomenon—that is all but patient-centric. When research is used as the sole instrument to get promoted and advance in careers, the quality and integrity of produced research can quickly become a non-priority. In oncology in under-resourced settings, where training is lacking or limited, such a risk appears to be substantially higher [2].

The African Organization for Research and Training in Cancer (AORTIC) has recently established a comprehensive training program on research integrity in oncology using online-based education approaches (see Supplement). These encompassed advanced courses that aimed to enhance the quality of cancer research across Africa by providing guidance to researchers to respond to the unmet need of global oncology initiatives while incorporating principles of research integrity. The program sessions included six advanced courses that discussed insights on predatory publishing, data manipulation and retraction, plagiarism and copyright concerns, “paper mills phenomenon” (i.e., companies creating fake data and writing scientific papers), gender inequity, and appraisal of clinical trials and meta-analyses beyond statistical significance.

The first advanced course was aimed at increasing awareness of publishing in predatory journals. This was an opportunity to discuss the critical value of peer-review in ensuring the quality and credibility of scientific publications in oncology. Moreover, participants in the course were also introduced to the characteristics of predatory and hijacked journals as well as their deceptive tactics used to attract researchers and bypass the peer-review system, which is a vital component of research. The discussion extended to the negative implications of predatory publishing in clinical practice and how their use as a source of oncology knowledge may harm patients with cancer. Insights from our research [3, 4] on how oncologists perceive predatory publishing and the challenges they face in navigating the complex landscape of academic publishing were also discussed. The session also explored the impact of educational interventions [3] to fight predatory journals and offered guidance and support for young researchers, including those who had inadvertently published in predatory journals, to enhance transparency and credibility of their research. The course concluded by emphasizing the potential of African oncology in promoting ethical open access, and therefore, an optimal dissemination of research outputs.

The critical issue of data manipulation and retractions in cancer research was developed in a second advanced course that offered basic knowledge to illustrate the severity of these unethical practices. Illustrative examples from the Retraction Watch database initiative [5] with real-world cases of data falsification in cancer research and the resulting harm caused by such practices were reviewed with participants with key take-home messages. The session highlighted the Committee on Publication Ethics (COPE) guidelines and their importance in guiding responsible research conduct [6]. A case study on retracted articles from African authors focused on the drivers of misconduct and provided valuable lessons and insights for young researchers. Solutions and recommendations to increase awareness about the danger of research fraud and its prevention were provided.

A third advanced course on plagiarism and copyright issues in cancer research provided participants with key and precise definitions and examples of what should be considered as plagiarism and cases of ethical reuse of previously published materials. This session explored illustrative cases from African oncology researchers. This was followed by a discussion of the results of a survey among researchers in Morocco that had shed light on factors and motivations behind plagiarism misconduct and its generalizability in other similar settings in Africa [7]. Practical solutions and guidance for preventing copyright concerns, plagiarism, and overlap in publications to avoid associated retractions were also addressed.

“Paper mill” factory is an emerging research misconduct in academia where many falsified papers are being discovered that evaded peer-reviewed journals [8]. Inter alia, paper mill threatens scholarly research integrity and engenders distrust in science, leads to false claims in positive results, promotes unethical academic promotion, and leads to reluctance to share data [8, 9]. A course on this problem of research integrity was delivered to increase awareness of fraudulent research papers resulting from this budding concern. This session also encompassed the implications of impaired disclosure of conflicts of interest in oncology. Indeed, pharmaceutical industry-oncologists relationships in sub-Saharan Africa are increasingly becoming more complex, with issues reported in disclosing potential conflicts of interest, and unclear benefit-harms tradeoff brought to the continent [10]. The course was also an opportunity to review the damages of the lack of ethical board review and informed consent in cancer research with examples of retractions linked to the absence of adhering to the Helsinki declaration of ethical standards.

Given the fact that gender inequity in oncology is a rising concern in Africa [1], the fifth advanced course presented a session on identifying gaps and barriers toward gender equity. Participants were provided with a comprehensive overview of the current state of women representation in cancer research, drawing data from all over the globe [11] and with a focus on Africa with a discussion of the results of two bibliometric studies conducted on North- and sub-Saharan African research outputs [1, 12]. This session also offered practical tools and approaches to promote gender equity in cancer research, highlighting the global efforts for inclusivity and diversity.

The final session challenged the reliance on statistical significance over clinical relevance in appraising the oncology literature. The course reviewed the impact of adherence to good practices when reading the results of phase III trials on patients’ outcomes. The program also examined the importance to report value metrics with validated tools, including the ESMO-Magnitude of Clinical Benefit Scale (ESMO-MCBS) scoring system and its advantages in reviewing biases in study design, implementation, and data analysis that may alter the appraisal of clinical benefits [13]; potential needs to enhance applicability for the African settings were highlighted. Furthermore, practical guidance was provided on how to critically appraise meta-analyses in oncology, including methods for identifying heterogeneity and addressing publication bias, and highlighting the importance of critical appraisal in maintaining research integrity. Ultimately, we supported the global endeavors and devotion to value-driven such as “Common Sense in Oncology”, for more patient-centered approaches and equitable cancer care [14].

The commitment of the AORTIC in enhancing cancer research in Africa is an illustrative example of how oncology societies can actively participate in the development of a competent oncology workforce. The supportive engagement with international partners to improve cancer research in Africa [15], the AORTIC vision and the efforts of the Lancet Commission on Cancer in sub-Saharan Africa [16] are collectively encouraging actions to facilitate career advancement and research capacity-building to empower African researchers, ensure local autonomy toward cancer control, and to mitigate inequities in African oncology research [17]. In the quest to achieve its goals, AORTIC is an example to be followed by other oncology societies in low- and middle-income countries to mutually engage in reducing disparities in cancer research through education and training. Investing in human development by training can be facilitated with smart solutions, including distance-based education approaches [3] which appear to be promising for settings with limited resources, such as many African countries.

## Supplementary information

AORTIC Webinar 1

## Data Availability

No datasets were generated or analyzed during the current study.

## References

[1] Mutebi M, Lewison G, Aggarwal A, Alatise OI, Booth C, Cira M, et al. Cancer research across Africa: a comparative bibliometric analysis. BMJ Glob Health. 2022;7:e009849. 10.1136/bmjgh-2022-009849. 36356985 10.1136/bmjgh-2022-009849 PMC9660667

[2] Pramesh CS, Badwe RA, Bhoo-Pathy N, Booth CM, Chinnaswamy G, Dare AJ, et al. Priorities for cancer research in low- and middle-income countries: a global perspective. Nat Med. 2022;28:649–57. 10.1038/s41591-022-01738-x. 35440716 10.1038/s41591-022-01738-x PMC9108683

[3] El Bairi K, Fourtassi M, El Fatimy R, El Kadmiri N. Distance education as a tool to improve researchers’ knowledge on predatory journals in countries with limited resources: the Moroccan experience. Int J Educ Integr. 2023;19:1–15.

[4] El Bairi K, Trapani D, Nidhamalddin SJ, Zeb Khan S, Chowdhury AR, Lengyel CG, et al. 1326P Oncology under attack by predatory journals: a global survey. Ann Oncol. 2022;33:S600–S615. 10.1016/annonc/annonc1069.

[5] https://retractionwatch.com/.

[6] Patel J. New COPE guidelines on publication process manipulation: why they matter. Res Integr Peer Rev. 2018;3:13. 10.1186/s41073-018-0059-x. 30505466 10.1186/s41073-018-0059-x PMC6260777

[7] El Bairi K, El Kadmiri N, Fourtassi M. Exploring scientific misconduct in Morocco based on an analysis of plagiarism perception in a cohort of 1,220 researchers and students. Account Res. 2022:1–20. 10.1080/08989621.2022.2110866. 10.1080/08989621.2022.2110866 35938392

[8] Candal-Pedreira C, Ross JS, Ruano-Ravina A, Egilman DS, Fernández E, Pérez-Ríos M. Retracted papers originating from paper mills: cross sectional study. BMJ. 2022;379:e071517. 10.1136/bmj-2022-071517. 36442874 10.1136/bmj-2022-071517 PMC9703783

[9] Heck S, Bianchini F, Souren NY, Wilhelm C, Ohl Y, Plass C. Fake data, paper mills, and their authors: The International Journal of Cancer reacts to this threat to scientific integrity. Int J Cancer. 2021;149:492–493. 10.1002/ijc.33604. 33905542 10.1002/ijc.33604

[10] Rubagumya F, Mutebi M, Manirakiza A, Abdihamid O, Mushonga M, Vanderpuye V, et al. Pharmaceutical industry relationships with oncologists in sub-Saharan Africa. Lancet Oncol. 2023;24:e96–e101. 10.1016/S1470-2045(22)00639-8. 36725154 10.1016/S1470-2045(22)00639-8

[11] Teuwen L-A, Young J, Davies A, Hudson J, Bourlon de los Rios MT, Prenen H, et al. Representation of countries and gender in abstracts at the 2022 American Society of Clinical Oncology Annual Scientific Meeting (ASCO ASM). Ann Oncol. 2022;33:S1609. 10.1016/j.annonc.2022.10.463.

[12] El Bairi K, GEORGiNA study collaborators. Systematic mapping of gender disparities in oncology publications of north African countries: the GEORGiNA study. JCO Glob Oncol. 2023;9:112–112. 10.1200/GO.2023.9.Supplement_1.112.

[13] Gyawali B, de Vries EGE, Dafni U, Amaral T, Barriuso J, Bogaerts J, et al. Biases in study design, implementation, and data analysis that distort the appraisal of clinical benefit and ESMO-Magnitude of Clinical Benefit Scale (ESMO-MCBS) scoring. ESMO Open. 2021;6:100117. 10.1016/j.esmoop.2021.100117. 33887690 10.1016/j.esmoop.2021.100117 PMC8086024

[14] Booth CM, Sengar M, Goodman A, Wilson B, Aggarwal A, Berry S, et al. Common Sense Oncology: outcomes that matter. Lancet Oncol. 2023;24:833–835. 10.1016/S1470-2045(23)00319-4. 37467768 10.1016/S1470-2045(23)00319-4

[15] Vogel AL, Freeman JA, Duncan K, Alaro J, Welch JJ, Rodrigues B, et al. Advancing cancer research in Africa Through Early-Career Awards: the BIG Cat initiative. J Glob Oncol. 2019;5:1–8. 10.1200/JGO.18.00223. 31009270 10.1200/JGO.18.00223 PMC6528731

[16] Ngwa W, Addai BW, Adewole I, Ainsworth V, Alaro J, Alatise OI, et al. Cancer in sub-Saharan Africa: a Lancet Oncology Commission. Lancet Oncol. 2022;23:e251–312. 10.1016/S1470-2045(21)00720-8. 35550267 10.1016/S1470-2045(21)00720-8 PMC9393090

[17] Rubagumya F, Carson L, Mushonga M, Manirakiza A, Murenzi G, Abdihamid O, et al. An analysis of the African cancer research ecosystem: tackling disparities. BMJ Glob Health. 2023;8:e011338. 10.1136/bmjgh-2022-011338. 36792229 10.1136/bmjgh-2022-011338 PMC9933677

