# Supplementary material for: Integrity matters in oncology: AORTIC takes a stand against research misconduct
Source: BJC Rep. 2024 Feb 12;2:11. doi: 10.1038/s44276-023-00032-8 (PMC11523996; doi:10.1038/s44276-023-00032-8)
Supplement: Supplementary file 1 — AORTIC Webinar 1 [file 44276_2023_32_MOESM1_ESM.pdf]

AORTIC
OAREC

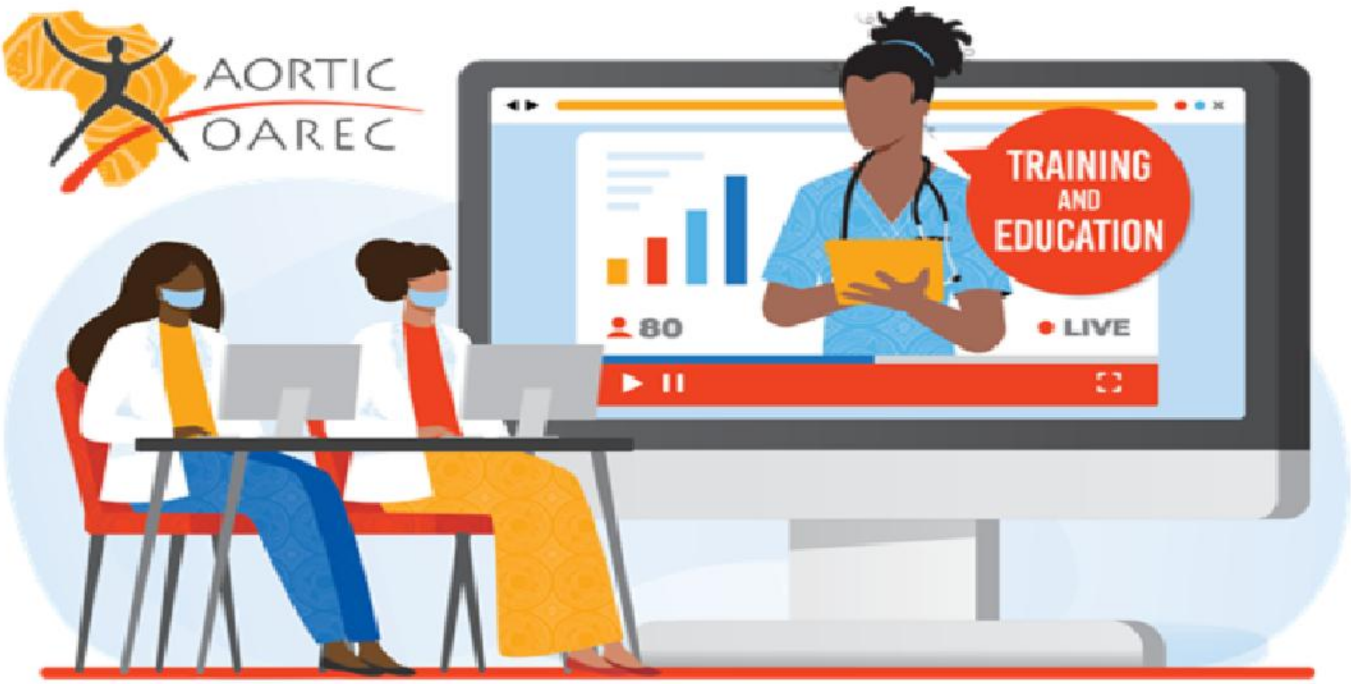

Webinar 1 of 6
"Predatory Publishing in Oncology: Say No to Evil"

Register here

Wednesday 12 July 2023

15h00 – 16h00 WAT / UTC +2 hours

MODERATOR

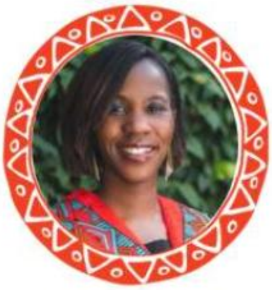

Miriam Mutebi
AORTIC President Elect
Aga Khan University Hospital
Kenya

SPEAKER

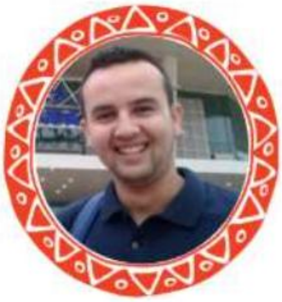

Khalid El Bairi
AORTIC Member &
Mohammed VI University Hospital
Morocco

PROGRAMME

- *Historical focus on the value of peer-review in oncology*
- *General features of predatory and hijacked journals*
- *The danger of predatory publishing in clinical practice*
- *Perception of oncologists on predatory publishing*
- *Educational interventions to increase awareness on predatory journals*
- *Guidelines/solutions to increase transparency of authors who published in predatory journals*
- *Africa is the future of ethical open access*
- *Personal perspectives: the hidden predatory agenda of non-predatory journals*

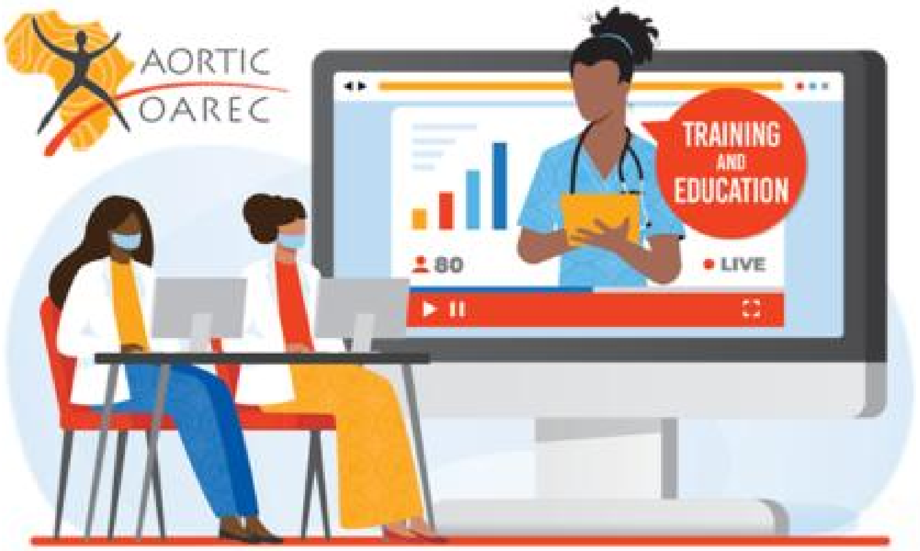

AORTIC Research Integrity Webinar Series - Part Two

Data Manipulation and Retractions in Cancer Research

RESEARCH INTEGRITY WEBINAR – 2 of 6

"Data Manipulation and Retractions in Cancer Research"

Wednesday 19 July 2023

16h00 - SAST (South Africa) |15h00 - WAT (Nigeria) & WEST (Morocco)| 17h00 - EAT (Kenya) & EEST (Egypt) | 14h00 - GMT (Ghana) | 10h00 - EDT (Washington USA) | 09h00 - PDT (Seattle USA)

MODERATOR

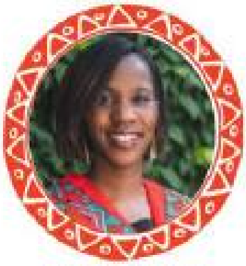

Miriam Mutebi
AORTIC President Elect
Aga Khan University Hospital
Kenya

SPEAKER

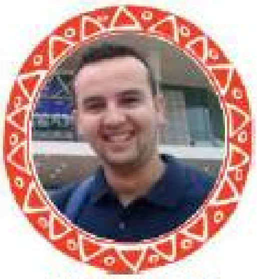

Khalid El Bairi
AORTIC Member &
Mohammed VI University Hospital
Morocco

PROGRAMME

- Data manipulation and falsifications: Some definitions.
- Illustrative examples of manipulated data in cancer research.
- Retractions in Oncology: Focus on COPE guidelines.
- The Retraction Watch Database initiative.
- Retracted articles in African Oncology: Some examples with take home messages.
- Solutions and recommendations to increased awareness on research fraud in oncology.

AORTIC
OAREC

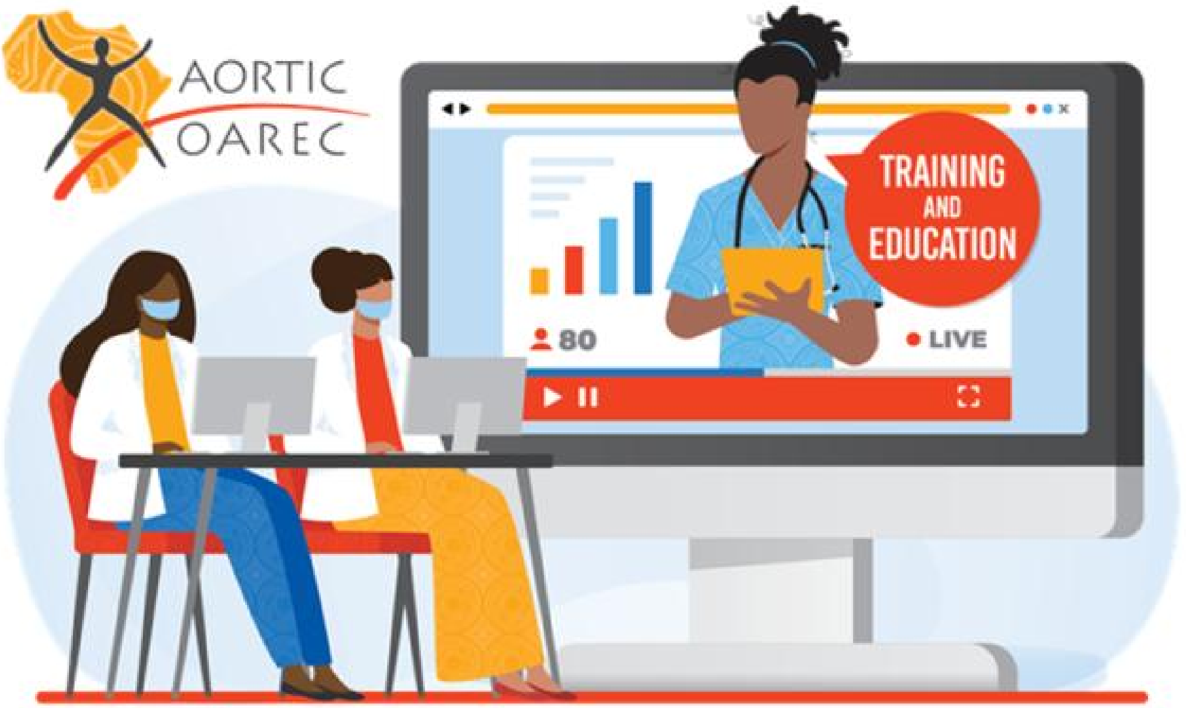

RESEARCH INTEGRITY WEBINAR – 3 of 6

"Plagiarism and Copyright Issues in Oncology"

Wednesday 15 August 2023

16h00 - SAST (South Africa) |**15h00 - WAT** (Nigeria) & **WEST** (Morocco)| **17h00 - EAT** (Kenya) & **EEST** (Egypt) | **14h00 - GMT** (Ghana) | **10h00 - EDT** (Washington USA) | **09h00 - PDT** (Seattle USA)

MODERATOR

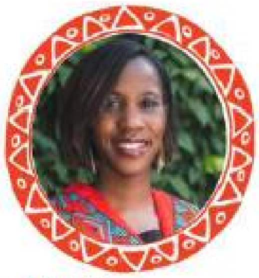

Miriam Mutebi
AORTIC President Elect
Aga Khan University Hospital
Kenya

SPEAKER

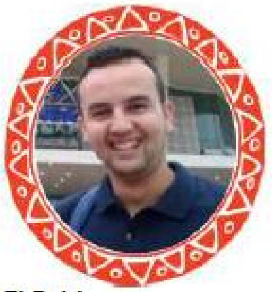

Khalid El Bairi
AORTIC Member &
Mohammed VI University Hospital
Morocco

PROGRAMME

- Plagiarism and overlap in oncology literature: Some key definitions and examples.
- Plagiarism in Africa: A focus on retracted articles from Retraction Watch database.
- Why do researchers plagiarize? A survey of Moroccan scholars.
- Solutions and recommendation to limit the issue of plagiarism.
- Copyright issues in academia: some examples and how to avoid associated retractions.

AORTIC
OAREC

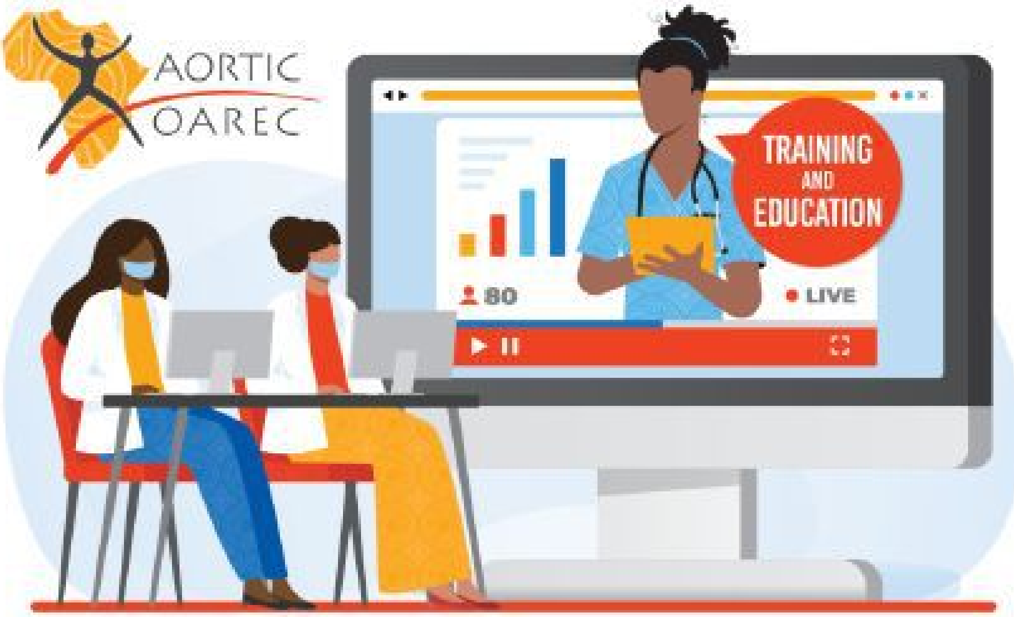

RESEARCH INTEGRITY WEBINAR- 4 OF 6

"The Fake Science Industry in Oncology and Other Issues in Research Integrity"

Thursday 7 September 2023

16h00 SAST (South Africa) |15h00 WAT (Nigeria) & (Morocco)

17h00 EAT (Kenya) & EEST (Egypt) | 14h00 GMT (Ghana)

10h00 EDT (Washington USA) | 09h00 PDT (Seattle USA)

MODERATOR

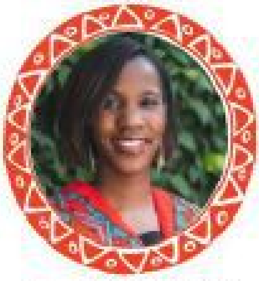

Miriam Mutebi
AORTIC President Elect
Aga Khan University Hospital
Kenya

SPEAKER

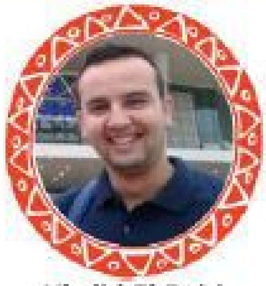

Khalid El Bairi
AORTIC Member &
Mohammed VI University Hospital
Morocco

PROGRAMME

1. *Overview of paper mill factory in oncology with illustrative examples*
2. *Impaired disclosure of conflicts of interests in oncology*
3. *Retractions in oncology associated with lack of ethics committee review and informed consents.*
4. *Take home messages for young oncologists*

AORTIC
OAREC

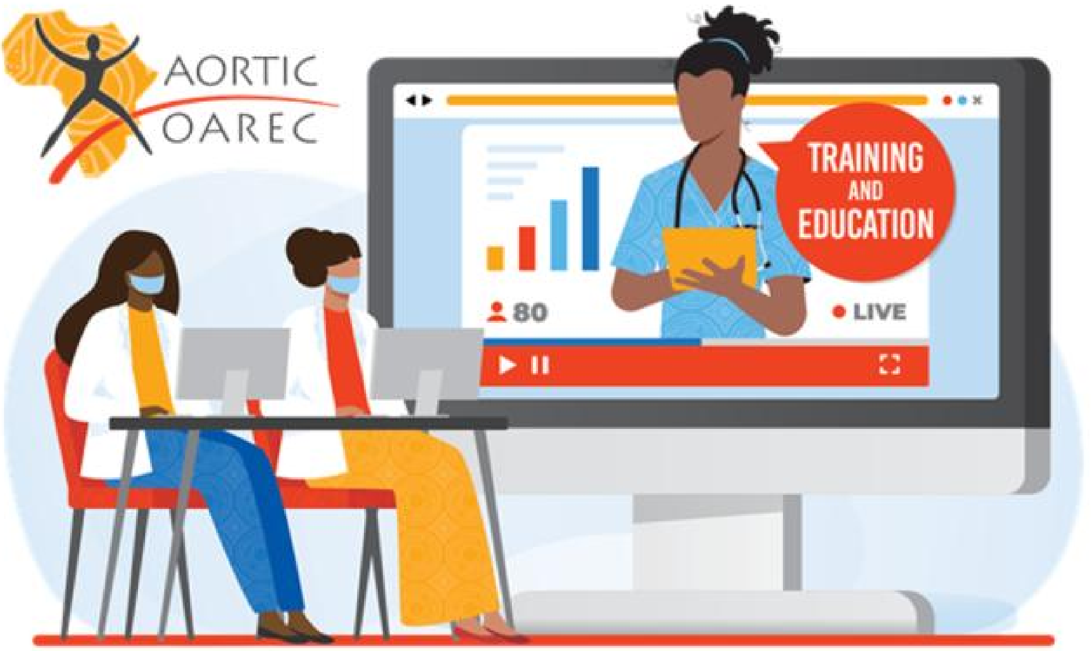

RESEARCH INTEGRITY WEBINAR- 5 OF 6

"Exploring Gender Inequity in Oncology: Toward More Diversity and Inclusion"

Wednesday 13 September 2023

16h00 SAST (South Africa) |15h00 WAT (Nigeria) & (Morocco)

17h00 EAT (Kenya) & EEST (Egypt) | 14h00 GMT (Ghana)

10h00 EDT (Washington USA) | 09h00 PDT (Seattle USA)

SPEAKERS

Dr Miriam Mutebi | Aga Khan University Hospital & AORTIC President Elect - Kenya

Khalid El Bairi | Mohammed VI University Hospital & AORTIC Member - Morocco

Dr Laure-Anne Teuwen | University Hospital Antwerp & AORTIC Member - Belgium

PROGRAMME

Representation of Women in Oncology: Toward Gender Equity?

1. Looking back: identified gaps and barriers | Dr Laure Anne Teuwen (Belgium) *5 mins*
2. The present: where are we at?
   - Europe/Asia: results from the ESMO women for oncology committee survey 2021 | Dr Laure Anne Teuwen (Belgium) *10 mins*
   - USA: Results from the ASCO 2022 phase 3 trial abstracts analysis | Dr Laure Anne Teuwen (Belgium)
   - North Africa: The GEORGiNA study | Dr Khalid El Bairi (Morocco) *5 mins*
   - Subsaharan Africa | Dr Miriam Mutebi (Kenya) *5 mins*
3. Towards the future: tools to increase gender equality | Dr Laure Anne Teuwen (Belgium) *5-10 mins*

AORTIC
OAREC

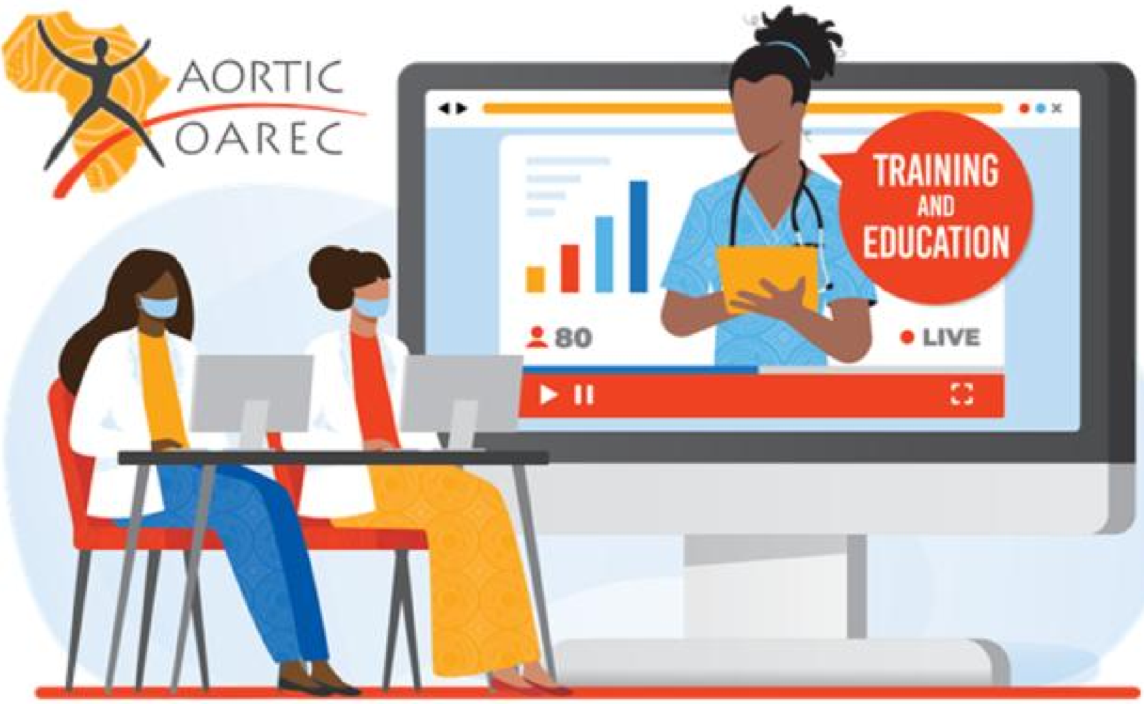

RESEARCH INTEGRITY WEBINAR- 6 OF 6

"Critical Appraisal of Oncology Literature: Beyond Statistical Significance"

Wednesday 20 September 2023

16h00 SAST (South Africa) |15h00 WAT (Nigeria) & (Morocco)

17h00 EAT (Kenya) & EEST (Egypt) | 14h00 GMT (Ghana)

10h00 EDT (Washington USA) | 09h00 PDT (Seattle USA)

SPEAKERS

Khalid El Bairi | Mohammed VI University Hospital & AORTIC Member - Morocco

Dario Trapani | European Institute of Oncology (IEO) - Italy

PROGRAMME

Dr Dario Trapani | Review of Phase III Trials in Oncology: Statistical Significance versus Clinical Relevance (Duration 15 minutes)

Dr Khalid El Bairi | How to Appraise a Meta-analysis of Randomized and Controlled Clinical Trials in Oncology (Duration 20 minutes)

- Quality overview of systematic reviews
- Reading a Forest plot
- How to identify heterogeneity in a meta-analysis
- Solutions for moderate and high heterogeneity
- Publication bias: the Funnel plot
- Illustrative examples through a clinical case
- Bad science in oncological meta-analyses
